# Application of leucine dehydrogenase Bcd from *Bacillus subtilis* for l-valine synthesis in *Escherichia coli* under microaerobic conditions

**DOI:** 10.1016/j.heliyon.2019.e01406

**Published:** 2019-04-04

**Authors:** Ekaterina A. Savrasova, Nataliya V. Stoynova

**Affiliations:** Ajinomoto-Genetika Research Institute, Moscow, 117545 Russia

**Keywords:** Biochemistry, Microbiology, Genetics

## Abstract

Microaerobic cultivation conditions have been shown experimentally and theoretically to improve the performance of a number of bioproduction systems. However, under these conditions, the production of l-valine by *Escherichia coli* is decreased mainly because of a redox cofactor imbalance and a decreased l-glutamate supply. The synthesis of one mole of l-valine from one mole of glucose generates two moles of NADH via glycolysis but consumes a total of two moles of NADPH, one in the ketol-acid reductoisomerase (KARI) reaction and the other in the regeneration of l-glutamate as an amino group donor for the branched-chain amino acid aminotransferase (BCAT) reaction. The improvement of l-valine synthesis under oxygen deprivation may be due to solving these problems. Increased l-valine synthesis under oxygen deprivation conditions was previously shown in *Corynebacterium glutamicum* (Hasegawa et al., 2012). In this study, we have proposed the use of NADH-dependent leucine dehydrogenase (LeuDH; EC 1.4.1.9) Bcd from *B. subtilis* instead of the native NADPH-dependent pathway including aminotransferase encoded by *ilvE* to improve l-valine production in *E. coli* under microaerobic conditions. We have created l-valine-producing strains on the base of the aminotransferase B-deficient strain V1 (B-7 Δ*ilvBN* Δ*ilvIH* Δ*ilvGME:*:P_L_*-ilvBN*^*N17K*^*DA*) by introducing one chromosomal copy of the *bcd* gene or the *ilvE* gene. Evaluation of the l-valine production by the obtained strains under microaerobic and aerobic conditions revealed that leucine dehydrogenase Bcd had a higher potential for l-valine production under microaerobic conditions. The Bcd-possessing strain exhibited 2.2-fold higher l-valine accumulation (up to 9.1 g/L) and 2.0-fold higher yield (up to 35.3%) under microaerobic conditions than the IlvE*-*possessing strain. The obtained results could be interpreted as follows: an altering of redox cofactor balance in the l-valine biosynthesis pathway increased the production and yield by *E. coli* cells under microaerobic conditions. Thus, the effective synthesis of l-valine by means of “valine fermentation” was shown in *E. coli*. This methodology has the advantages of being an economical and environmentally friendly process.

## 1 Introduction

**Fig. 1:**
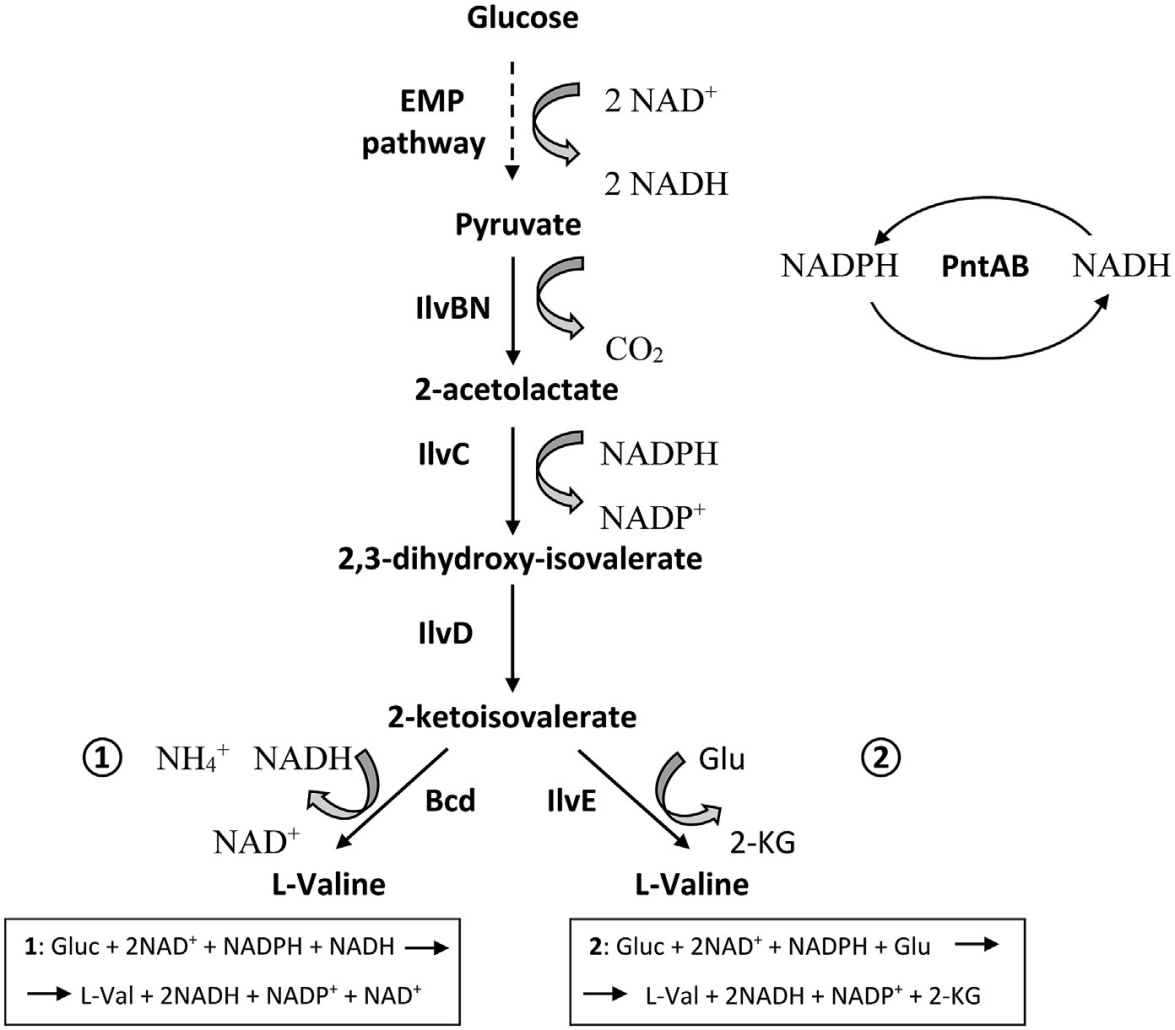
Pathway of l-valine biosynthesis. EMP pathway, Embden-Meyerhof-Parnas pathway; IivBN, acetolactate synthase I; IlvC, ketol-acid reductoisomerase; IlvD, dihydroxyacid dehydratase; IlvE, branched-chain amino acid aminotransferase; Bcd, leucine dehydrogenase; PntAB, pyridine nucleotide transhydrogenase.

l-Valine (hereinafter, valine) is a branched-chain amino acid (BCAA) that is widely used in dietary products, pharmaceuticals, and cosmetics, as an animal feed additive and as a precursor in the chemical synthesis of antibiotics and herbicides (Park and Lee, 2010). In addition, an immediate precursor of valine, 2-ketoisovalerate (Fig. 1), is an initial compound in the biosynthesis of isobutanol, a promising biofuel (Atsumi et al., 2008; Savrasova et al., 2011). To date, valine has been produced by microbial synthesis, mainly by using engineered *E. coli* and *Corynebacterium glutamicum.* General strategies to develop efficient valine-producing strains have been reported (Blombach et al., 2008; Park et al., 2007, 2011; Park and Lee, 2010; Wang et al., 2018).

Usually, amino acids are produced from sugars by microbes during aerobic cultivation. Particularly for *E. coli* cells, aerobic conditions are preferable from the viewpoint of cell energetic and growth rate. Oxygen is an effective electronic acceptor and can provide a significantly higher ATP/glucose yield (more than 30 ATP per glucose under aerobic conditions vs only 2 ATP from the glycolysis pathway under anaerobic conditions). However, in some cases, anaerobic cultivation may increase the product yield, as *E. coli* is a metabolically versatile bacterium able to respond to changes in oxygen availability. This approach exploits a flexible biochemistry in which aerobic respiration is preferred to anaerobic respiration, which in turn is preferred to fermentation (Partridge et al., 2007). *E. coli* cells can be intentionally adapted to microaerobic conditions, *e.g*., by laboratory adaptive evolution (Partridge et al., 2007).

Microaerobic conditions have been shown experimentally and theoretically to improve the performance of a number of bioproduction systems. However, under oxygen deprivation conditions, NADH is oxidized in mixed fermentation pathways, resulting in ethanol, acetate, lactate, and succinate production (Böck and Sawers, 1996); otherwise, excess NADH inhibits glycolysis, particularly its NAD^+^-dependent glyceraldehyde-3-phosphate dehydrogenase reaction. In this respect, the potential use of NADH formed under microaerobic conditions as a driving force for synthesis of a target compound is of particular interest. The microbial production of valine seems to be one of the most appropriate models. Previously, the production of different compounds (ethanol, lactate, succinate, organic acid, l-alanine, l-valine) by *C. glutamicum* was shown under oxygen deprivation conditions (Hasegawa et al., 2012, 2013; Inui et al., 2004a, 2004b, 2007; Jojima et al., 2010). In addition, improved isobutanol synthesis in *E. coli* under anaerobic conditions via the NADH-dependent pathway was shown (Bastian et al., 2011).

In some cases, *E. coli* is preferable over *C. glutamicum* as a host for the microbial production of useful compounds because of faster cell growth and better developed genetic tools. In this work, we demonstrated the effective synthesis of valine by means of so-called “valine fermentation” under microaerobic conditions (Fig. 1).

**Fig. 2:**
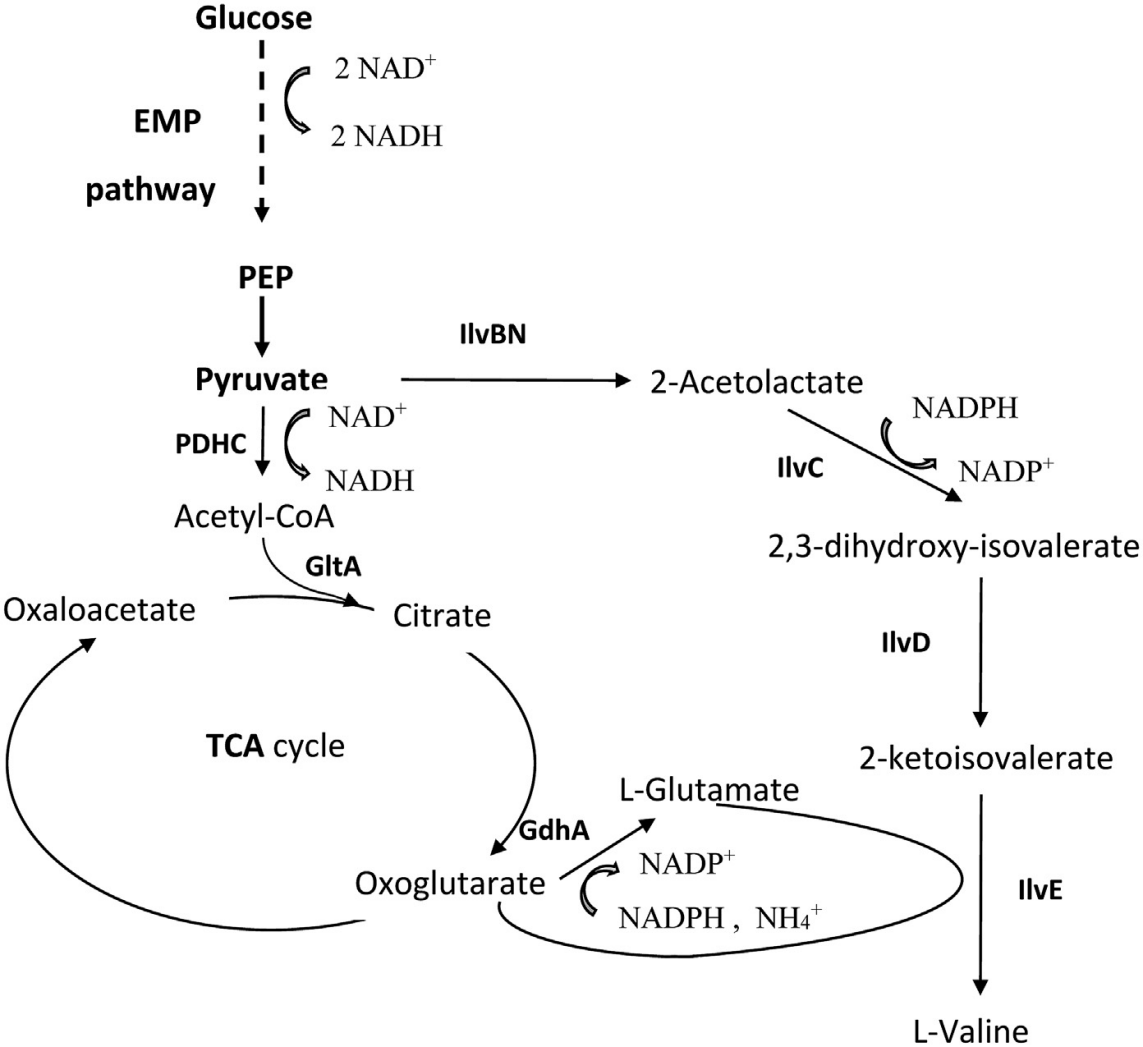
Metabolic pathway of *E. coli* and l-valine biosynthetic pathway. EMP pathway, Embden–Meyerhof–Parnas pathway; PDHC, pyruvate dehydrogenase complex; GltA, citrate synthase; IivBN, acetolactate synthase I; IlvC, ketol-acid reductoisomerase; IlvD, dihydroxyacid dehydratase; IlvE, branched-chain amino acid aminotransferase; GdhA, glutamate dehydrogenase.

The valine biosynthetic pathway in *E. coli* consists of four reactions catalyzed by enzymes (Fig. 2): acetohydroxy acid synthase, which catalyzes the first common step in BCAA biosynthesis (isoenzymes AHAS I, II, III, encoded by *ilvBN*, *ilvGM*, and *ilvIH,* respectively); ketol-acid reductoisomerase (KARI), encoded by *ilvC*; dihydroxy acid dehydratase (DHAD), encoded by *ilvD*; and BCAA aminotransferase (BCAT, hereinafter AT), encoded by *ilvE*. The pathway is also responsible for the biosynthesis of other BCAAs (l-leucine and l-isoleucine) and D-pantothenate (Park et al., 2007; Park and Lee, 2010). The key enzyme among the four is AHAS because it is subject to different regulation (Umbarger, 1996). Expression of the *ilvGMEDA* operon is controlled by transcriptional attenuation mediated by all three BCAAs (Lawther and Hatfield, 1980; Lawther et al., 1987), whereas the *ilvBN* operon is controlled by attenuation mediated only by l-valine and l-leucine (Umbarger, 1996; Wek et al., 1985). In this work, we have created *E. coli* valine-producing strains containing feedback-resistant AHAS I encoded by the *ilvBN*^*N17K*^ genes as a part of the artificial operon P_L_*-ilvBN*^*N17K*^*DA* in the chromosome (Sycheva et al., 2009).

The biosynthesis of one mole of valine requires two moles of NADPH, one of which is consumed in the glutamate dehydrogenase (GDH; EC 1.4.1.4) (Sakamoto et al., 1975; Helling, 1994) reaction, which yields l-glutamate as a universal amino group donor for aminotransferase reactions, including the BCAA aminotransferase (BCAT; EC 2.6.1.42)-mediated (Inoue et al., 1988) synthesis of valine from its immediate keto precursor (Fig. 2). As an alternative, valine synthesis can proceed via the NADH-dependent BCAA dehydrogenase reaction, thereby providing the NADH oxidation necessary for the function of the Embden-Meyerhof-Parnas (EMP) pathway yielding pyruvate, a starting compound for valine synthesis. A range of microorganisms other than *E. coli* possess such dehydrogenases, and NADH-specific BCAA dehydrogenase (leucine dehydrogenase; LeuDH; EC 1.4.1.9) enables the reversible reductive amination of BCAA keto precursors using ammonia directly as a substrate (Livesey and Lund, 1988; Nagata et al., 1988; Ohshima et al., 1994; Debarbouille et al., 1999).

In the case of valine synthesis by *E. coli* under oxygen deprivation conditions via the native metabolic pathway, including the aminotransferase reaction, the redox cofactor imbalance - two moles of NADH are synthesized via the EMP pathway, and two moles of NADPH are consumed in valine synthesis - should be overcome by the native enzyme systems of a cell (Fig. 1). There are various strategies to solve this problem. One of the methods is changing the cofactor requirement of valine biosynthetic reactions from NADPH to NADH to improve the redox status of a cell. Alternatively, a heterologous BCAA dehydrogenase can be used to provide NADH-dependent branched-chain keto acid amination instead of ordinary NADPH-dependent metabolic pathway, including the BCAA aminotransferase reaction (Fig. 1). In this case, glycolysis is simultaneously promoted by NADH oxidation due to so-called “valine fermentation” by analogy with traditional fermentation types, such as ethanol fermentation, lactate fermentation, etc. Additionally, the change in KARI cofactor specificity from NADPH to NADH is also useful, as was realized earlier for *C. glutamicum* (Hasegawa et al., 2012, 2013). However, for *E. coli*, this approach is affected by the presence of a membrane-bound pyridine nucleotide transhydrogenase PntAB, which catalyzes the energy-dependent transfer of reducing power from NADH to NADP^+^ (Sauer et al., 2004). In contrast, *C. glutamicum* does not possess a chromosomally encoded nicotinamide nucleotide transhydrogenase to catalyze the reversible interconversion between NADH and NADPH (Kabus et al., 2007), and NADPH formation from NADH via malic enzyme would play only a minor role (Bartek et al., 2010; Georgi et al., 2005).

In this work, we showed an increase in valine production in *E. coli* under microaerobic conditions due to the improvement of the redox cofactor balance (NADH synthesis/NADPH consumption) by the introduction of NAD-specific LeuDH from *B. subtilis* encoded by the *bcd* gene instead of AT encoded by *ilvE*. Under these conditions, we achieved a 2.2-fold increase in valine production and a 2.0-fold increase in yield (m/m) compared to the use of the traditional metabolic pathway including the BCAA aminotransferase reaction.

## 2 Materials and methods

### 2.1 Strains and media

All bacterial strains and plasmids used in this study are listed in Table 1. The XL1-Blue *E. coli* strain was used for cloning. The CC118 *(*λpir^+^) *E. coli* strain was used for the maintenance of *pir*-dependent recombinant plasmids. The following media were used for bacteria culture: lysogenic broth (LB), SOB, SOC and M9 medium (Sambrook and Russell, 2001). Glucose (0.4%) was added to minimal media as a carbon source. Ampicillin (Ap, 100 mg/L), chloramphenicol (Cm, 20 mg/L), tetracycline (Tc, 20 mg/L) and kanamycin (Km, 40 mg/L), were used for selection as necessary. Cultivation of valine-producing strains was carried out in fermentation medium (FM) at pH 7.0 containing the following (g/L): (NH_4_)_2_SO_4_, 15; KH_2_PO_4_, 1.5; MgSO_4_, 1; CaCO_3_, 20; B1, 0.01; glucose, 60.

**Table 1:** Bacterial strains and plasmids used in present study.

Strain or plasmid	Relevant genotype	Source or reference
Strains		
XL-Blue	*E. coli* (*recA1 endA1* gyrA96 (Nal^R^) *thi*-*1 hsdR17* (r_k_^_^ m_k_^_^)*glnV44 relA1 lac* [*F*’::Tn*10*(Tet^R^)*'proAB lacI*^*q*^ Δ(*lacZ*)*M15*])	Stratagene
CC118 *(*λpir^+^)	*E. coli* Δ(*ara-leu*) *araD* Δ*lacX74 galE galK phoA20 thi-1 rpsE rpoB argE*(Am) *recAl*, lysogenized with λpir phage	Herrero et al. (1990)
MG1655	*E. coli K12* wild-type	VKPM B-6195
MG1655 (Δφ80-*attB*_native_)φ80-*attB* trs5-7	*E. coli* K-12 MG1655 with deleted native φ80-*attB* site and φ80-*attB* in trs5-7 locus	Minaeva et al. (2008)
MG1655 4Δ	MG1655 Δ*ilvE* Δ*tyrB* Δ*avtA*::Km^R^ Δ*aspC*::Tet^R^	Laboratory collection
BW25113 Δ*ilvE::*Km^R^	*E. coli K12* Δ(*araD-araB*)*567* Δ(*rhaD-rhaB*)*568 ΔlacZ4787* (::rrnB-3) *hsdR514 rph-1 lacI*^+^ Δ*ilvE::*FRT*-kan-*FRT	Keio Collection
		Baba et al. (2006)Grenier et al. (2014)
MG1655 trs5-7::*bcd-*Tet^R^	MG1655 (Δφ80-*attB*_native_) trs5-7::*bcd*-λ*attL*-Tet^R^- λ*attR*	Present study
MG1655 trs5-7::*bcd*	MG1655 (Δφ80-*attB*_native_) trs5-7::*bcd* -Tet^S^	Present study
MG1655 trs5-7::*bcd* Δ*ilvE::*Km^R^	MG1655 (Δφ80-*attB*_native_) trs5-7::*bcd* -Tet^S^ Δ*ilvE*:: FRT*-kan-*FRT	Present study
MG1655 *cat*-P_L_-*bcd*_5.7_	MG1655 (Δφ80-*attB*_native_) trs5-7:: λ*attL*-Cm^R^- λ*attR*-P_L_-*bcd*	Present study
MG1655 *cat*-P_L_-*ilvE*_5.7_	MG1655 (Δφ80-*attB*_native_) trs5-7:: λ*attL*-Cm^R^- λ*attR*- P_L_-*ilvE* Δ*ilvE*:: FRT*-kan-*FRT	Present study
B-7 Δ*ilvBN* Δ*ilvGM* Δ*ilvIH*	*E. coli K12* Δ*ilvBN* Δ*ilvGM* Δ*ilvIH*	Sycheva et al. (2009)
B-7 Δ*ilvIH* Δ*ilvGM cat*-P_L_*-ilvBN*^*N17K*^	*E. coli K12* Δ*ilvIH* Δ*ilvGM* λ*attL*-Cm^R^- λ*attR*-P_L_- *ilvBN*^*N17K*^	Sycheva et al. (2009)
MG1655 *cat*-P_L_*-ilvBN*^*N17K*^*DA*	MG1655Δ*ilvGME::*λ*attL*-Cm^R^- λ*attR*-P_L_- *ilvBN*^*N17K*^*DA*	Laboratory collection, Serebrianyi
B-7 Δ*ilvBN* Δ*ilvGM* Δ*ilvIH cat*-P_L_*-ilvBN*^*N17K*^*DA*	*E. coli K12* Δ*ilvIH* Δ*ilvBN* Δ*ilvGME*:: λ*attL*-Cm^R^- λ*attR*-P_L_-*ilvBN*^*N17K*^*DA*	Present study
V1	*E. coli K12* Δ*ilvIH* Δ*ilvBN* Δ*ilvGME*::P_L_-*ilvBN*^*N17K*^*DA-*Cm^S^	Present study
V1 *cat*-P_L_-*bcd*_5.7_	*E. coli K12* Δ*ilvIH* Δ*ilvBN*	Present study
	Δ*ilvGME*::P_L_-*ilvBN*^*N17K*^*DA* trs5-7:: λ*attL*-Cm^R^- λ*attR*-P_L_-*bcd*	
V1 *cat*-P_L_-*ilvE*_5.7_	*E. coli K12* Δ*ilvIH* Δ*ilvBN* Δ*ilvGME::*P_L_-*ilvBN*^*N17K*^*DA* trs5-7:: λ*attL*-Cm^R^- λ*attR*- P_L_-*ilvE*	Present study
Plasmids		
pUC57-bcd-Bsub	pMB1 ori; Amp^R^; *bcd*	Present study
pMW118	pSC101 ori; Amp^R^; MCS	GenBank accession number AB005475
pMW118-bcd	pSC101 ori; Amp^R^; P_lac_-*bcd*	Present study
pAH162- λ*attL*-Tet^R^- λ*attR* -2Ter	oriR; λ*attL*-Tet^R^- λ*attR*; *attP* φ80; MCS	Minaeva et al. (2008)
pAH162- λ*attL*-Tet^R^- λ*attR*-2Ter-bcd	oriR; λ*attL*-Tet^R^- λ*attR*; *attP* φ80; *bcd*	Present study
pAH123	oriR101, repA101ts, λcIts857, λP_R_→φ80-*int*, Amp^R^	Haldimann and Wanner (2001), GenBank accession number AY048726
pKD46	oriR101, repA101ts, *araC*, P_*araB*_-[γ, β, exo of phage λ], Amp^R^	Datsenko and Wanner (2000), GenBank accession number AY048746
pMW-Int-Xis	oriR101, repA101ts, λcIts857, λP_R_→λ*xis-int*, Amp^R^	Minaeva et al. (2008)

### 2.2 Cultivation conditions

Cells were preseeded in test tubes containing 3 ml of LB medium and incubated at 32 °C for 3 h on a rotary shaker (250 rpm). The preseeded cultures were then diluted 1:20 into 2 ml of FM medium in 20 × 200-mm test tubes. Strains V1 *cat*-P_L_-*bcd*_5.7_ and V1 *cat-*P_L_*-ilvE*_5.7_ were cultivated at 32 °C for 68 h on a rotary shaker (250 rpm). To provide microaerobic conditions, rubber stoppers were used instead of cotton stoppers.

### 2.3 Determination of amino acid and glucose concentrations

Accumulated valine was measured by thin-layer chromatography (TLC). TLC plates (10 х 15) cm were coated with 0.11-mm layers of Sorbfil silica gel containing no fluorescent indicator (Stock Company Sorbpolymer, Krasnodar, Russia). The Sorbfil plates were developed with a mobile phase consisting of isopropanol-ethylacetate-25% aqueous ammonia-water (16:16:5:10 v/v). A solution of ninhydrin (2% w/v) in acetone was used as the visualizing reagent. Residual glucose was measured by a Biosen glucose and lactate analyzer (EKF Diagnostics, UK).

### 2.4 DNA handling procedures

Protocols for the genetic manipulation of *E. coli* and techniques for isolating and manipulating nucleic acids were described previously (Sambrook and Russell, 2001). Restriction enzymes, T4 DNA ligase, High Fidelity PCR Enzyme Mix and 1-kb DNA Ladder were purchased from Thermo Scientific Inc (USA). Plasmids and genomic DNA were isolated using QIAGEN Plasmid Mini Kits (QIAGEN GmbH, Germany) and Bacterial Genomic DNA Kits (Sigma, USA), respectively. QIAquick Gel Extraction Kits (QIAGEN GmbH, Germany) were used to isolate DNA from agarose gels. Oligonucleotides were purchased from Sintol (Russia). The sequences of oligonucleotide primers are presented in Table 2. Synthesis of the *bcd* gene was performed by Sloning BioTechnology GmbH (Germany).

**Table 2:** Oligonucleotides used in this study.

Primers	Sequence (5′- 3′)	Purpose
P1	AAAGGATGAACTACGAGGAAGGGAACAACATTC-ATACGCTCAAGTTAGTATAAAAAAGCTGAAC	creation of *cat*-P_L_*-ilvBN*^*N17K*^
P2	ATGGGACGGTGCGTGCCGTCCCATTTTTTGTATTTACTGAAAAAACACCGCGATCTTGTTAAAC	creation of *cat*-P_L_*-ilvBN*^*N17K*^
P3	GTAAAGCGCTTACGCGTCGA	verification of *cat*-P_L_*-ilvBN*^*N17K*^ integration
P4	TGCAAGTGAAGTTGAGTTGTTC	verification of *cat*-P_L_*-ilvBN*^*N17K*^ integration
P5	GAATGATATCCATATCCTCGAC	verification of *bcd* integration
P6	GTCTTCTCACGGGAACGGTT	verification of *bcd* integration
P7	CGAAAGTGATTGCGCCTACCCGGATATTATCGTGAGCGCTCAAGTTAGTATAAAAAAGCTGAAC	creation of *cat*-P_L_ upstream *bcd* and *ilvE*
P8	TATATTTAAAAAGTTCCATACATAGATCTCCTTCTTCGGCCAATGCTTCGTTTCGTATCACACA	creation of *cat*-P_L_ upstream *bcd*
P9	CCATATTACGACCATGAGTGG	verification of *ilvE* deletion
P10	CCAGTAATTCAGAAATGTTGG	verification of *ilvE* deletion
P11	AGATAGATCTCCTTCTTCGGCCAATGCTTC	creation of *cat*-P_L_ upstream of *ilvE*
P12	ATTGGCCGAAGAAGGAGATCTATCTATGACCACGAAGAAAGCTGATTACATTT	amplification of *ilvE*
P13	AAGCTTGCATGCCTGCAGGTCGACTCTAGAGGATCCTTATTGATTAACTTGATCTAACCAGCCC	amplification of *ilvE*
P14	GTTCGTTGCAACAAATTGATAAG	sequencing of P_L_*-ilvE*
P15	CAGGGAAGAGAGGTAGTTACC	sequencing of P_L_*-ilvE*
P16	GATCGATGCGATGGTTTCCTC	sequencing of P_L_*-ilvE*

### 2.5 Plasmid construction

All plasmids used or constructed in this study are listed in Table 1.

#### 2.5.1 Construction of pUC57-bcd-Bsub

To express leucine dehydrogenase Bcd from *B. subtilis* in *E. coli*, the rare codon-free variant of the *bcd* gene was synthesized. To clone *bcd*, a *bcd* gene (GenBank accession number BSU24080) from *B. subtilis* with a modified nucleotide sequence codon-optimized for *E. coli* and with *Sac*I and *BamH*I restriction sites (5′**-**GAGCTCAAGAAGGAGATCTATGT**-**3′, 5′**-**GGATCC**-**3′) was synthesized by Sloning BioTechnology. In the modified *bcd* sequence, the following 29 codons were optimized: 8 Arg positions, 43, 59, 62, 108, 155, 341, 349 and 362 (codons AGA (7) and CGG (1) were replaced with CGC); 9 Gly positions, 23, 41, 78, 104, 143, 156, 166, 172 and 195 (codon GGA was replaced with GGC); 4 Pro positions, 137, 147, 222 and 329 (codons CCA (1) and CCT (3) were replaced with CCG); and 8 Thr positions, 33, 46, 80, 117, 129, 133, 149 and 266 (codon ACA was replaced with ACC). The resulting DNA fragment containing the *bcd* gene was digested with *Sac*I and *BamH*I and cloned into the pUC57 vector (GenBank accession number Y14837) cut with the same enzymes, yielding the plasmid pUC57-bcd-Bsub. The *bcd* gene was cloned in the opposite orientation relative to the Lac promoter to reduce the potential toxicity of the gene expression.

#### 2.5.2 Construction of pMW118-bcd

To clone *bcd*, pUC57-bcd-Bsub was digested with *Sac*I and *BamH*I. The DNA fragment containing the *bcd* gene was cloned into the pMW118 vector (under control of the P_lac_ promoter) and cut with the same enzymes, creating pMW118-bcd. The resulting plasmid was shown to complement the Val and Ile auxotrophy of the aminotransferase-deficient strain MG1655 4Δ under IPTG induction during growth on M9 minimal medium supplemented with appropriate amino acids.

#### 2.5.3 Construction of pAH162-λattL-Tet^R^-λattR-2Ter-bcd

The construction of the integrative vector pAH162 λ*attL*-Tet^R^-λ*attR*-2Ter was previously described (Minaeva et al., 2008). To clone *bcd*, pUC57-bcd-Bsub was digested with *Sac*I and *BamH*I. The DNA fragment containing the promoter-less *bcd* gene was cloned into the integrative vector pAH162-λ*attL*-Tet^R^-λ*attR*-2Ter cut with the same enzymes, yielding pAH162-λ*attL*-Tet^R^-λ*attR*-2Ter-bcd.

### 2.6 Strain construction

The primers and strains used and constructed in this study are listed in Tables 1 and 2. Chromosomal gene deletions and insertions in the chromosome of the *E. coli* strain MG1655 K-12 were prepared via the method developed by Datsenko and Wanner called “λRed-mediated recombination” (Datsenko and Wanner, 2000) combined with the phage λ Int/Xis-mediated marker excision. The plasmid pKD46 carrying the arabinose-inducible λ-Red genes was used to provide λ-Red recombination. φ80-Mediated integration was carried out according to (Haldimann and Wanner, 2001; Minaeva et al., 2008). The CRIM helper plasmid pAH123 containing the thermoinducible φ80-Int gene was used to provide φ80-mediated integration (Haldimann and Wanner, 2001). Specifically designed cassettes with the CmR^ex^ marker were transferred into the *E. coli* strains by P1 phage-mediated transduction (Miller, 1972). The DNA fragments CmR^ex^ and TetR^ex^ flanked by λ*attL/R* were eliminated from the *E. coli* chromosome using a λ-Int/Xis site-specific recombination system with the pMWts-λInt/Xis helper plasmid (Minaeva et al., 2008).

#### 2.6.1 Construction of the *E. coli* strain MG1655 ΔilvGME::λattL-Cm^R^-λattR-P_L_-ilvBN^N17K^DA

The artificial *ilv* operon *cat*-P_L_*-ilvBN*^*N17K*^*DA* was created by introducing the PCR fragment *cat*-P_L_-*ilvBN*^*N17K*^ to replace the *ilvGME* genes of the *ilvGMEDA* operon in the *E. coli* strain MG1655 via λ Red*-*mediated recombination (Datsenko and Wanner, 2000). The PCR fragment *cat*-P_L_-*ilvBN*^*N17K*^ (3.97 kbp) was created with primers P1 and P2 and the chromosome of strain B-7Δ*ilvGM*Δ*ilvIH* λ*attL*-Cm^R^-λ*attR*-P_L_-*ilvBN*^*N17K*^ as the template (Sycheva et al., 2009). The obtaining DNA fragment was introduced by electroporation into strain MG1655/pKD46, resulting in the strain MG1655 Δ*ilvGME::*λ*attL*-Cm^R^-λ*attR*-P_L_*-ilvBN*^*N17K*^*DA* (MG1655 *cat*-P_L_*-ilvBN*^*N17K*^*DA*). Integration was verified using primers P3 and P4.

#### 2.6.2 Construction of *E. coli* strain MG1655 trs5-7::λattL-Cm^R^-λattR-P_L_-bcd

The plasmid pAH162 λ*attL*-Tc^R^-λ*attR-*2Ter-bcd was integrated by the φ80-Int system into MG1655 φ80-*attB* trs5-7 Δφ80-*attB*_native_ using the Tc^R^ marker for selection to obtain the strain MG1655 trs5-7::λ*attL*-Tc^R^-λ*attR-bcd*. Integration was verified by using primers P5 and P6. The vector part of the integrated plasmid containing the Tc^R^ marker was excised by means of the pMWts-λInt/Xis helper plasmid, and the strain MG1655 trs5-7::*bcd* was obtained. The modification was verified by using primers P5 and P6. After that, the λ phage P_L_ promoter marked with Cm^ex^ upstream of the *bcd* gene was introduced by λ Red*-*mediated recombination (Datsenko and Wanner, 2000). The PCR fragment 1.97 kb containing the modification λ*attL*-Cm^R^-λ*attR-*P_L_ was created by using primers P7 and P8 with the chromosome of MG1655 λ*attL*-Cm^R^-λ*attR*-P_L_-*leuABCD* as a template. The obtained DNA fragment was introduced by electroporation into MG1655 trs5-7::*bcd*/pKD46, resulting in the strain MG1655 trs5-7::λ*attL*-Cm^R^-λ*attR*-P_L_-*bcd* (MG1655 trs5-7::*cat*-P_L_-*bcd*). The modification was checked by using primers P5 and P6.

#### 2.6.3 Construction of *E. coli* strain MG1655 trs5-7::λattL-Cm^R^-λattR-P_L_-ilvE ΔilvE::FRT-kan-FRT

To create an aminotransferase-overexpressing strain, we used the strain MG1655 trs5-7::*bcd* Δφ80-*attB*_native_ mentioned above. First, the introduction of *ilvE* deletion (Keio collection) marked with KmR^ex^ by P1 transduction was performed. The presence of this deletion was verified with primers P9 and P10. Then, the PCR fragment λ*attL*-Cm^R^-λ*attR*-P_L_-*ilvE* was introduced into this strain instead of the *bcd* gene by λ Red*-*mediated recombination (Datsenko and Wanner, 2000). The PCR fragment λ*attL*-Cm^R^-λ*attR*-P_L_-*ilvE*_5.7_ was created by overlap extension PCR with primers P7 and P13 by using the PCR fragments λ*attL*-Cm^R^-λ*attR*-P_L_ (created with primers P7 and P11) and *ilvE* (created with primers P12 and P13) as templates. The resulting 2.9 kb DNA fragment λ*attL*-Cm^R^-λ*attR*-P_L_-*ilvE*_5.7_ was introduced by electroporation into the strain MG1655 trs5-7::*bcd* Δ*ilvE*::FRT*-kan-*FRT/pKD46. The selection of integrants was performed on M9 minimal medium with 0.4% glucose to obtain the strain MG1655 trs5-7::λ*attL*-Cm^R^-λ*attR*-P_L_-*ilvE* Δ*ilvE*::FRT*-kan-*FRT (MG1655 trs5-7::*cat*-P_L_-*ilvE*) containing the P_L_ promoter marked with CmR^ex^ upstream of *ilvE*. The modification was checked by using primers P6 and P13. The obtained structure λ*attL*-Cm^R^-λ*attR*-P_L_-*ilvE*_5.7_ was verified by sequencing with primers P14, P15, and P16.

### 2.7 Preparation of cell extracts

Strains MG1655 *cat*-P_L_-*bcd*_5.7_ and MG1655 *cat-*P_L_*-ilvE*_5.7_ Δ*ilvE::*Km^R^ were cultured overnight, and 0.1 ml was used to inoculate fresh medium (10 ml of M9 medium supplemented with 1/10 v/v LB). Inoculated cultures were grown for 4.5 h until an optical density at 540 nm of 0.8 was reached. Cells were harvested by centrifugation and washed twice in 1 M sodium chloride and then in 0.1 M potassium–phosphate buffer (pH 7.0). Cells were suspended in 0.1 M potassium–phosphate buffer (pH 7.0) and disrupted by sonication. The supernatant after centrifugation at 12,000 rpm for 30 min was used as the cell extract. All steps were performed at temperatures <4 °C. Protein concentrations were determined by the Bradford assay (Bio-Rad protein assay, GmbH).

### 2.8 Branched-chain amino acid aminotransferase assay

Cell extracts were incubated for 15 min at 37 °C in 2-ml vials at pH 7.5 [0.5 M Tris hydrochloride (HCl) buffer + 1 mM dithiothreitol (DTT)] with 50 mM l-Phe and 50 mM 2-ketoisovalerate as the substrate and with 0.5 mM pyridoxal phosphate (PLP) as a cofactor in a total volume of 200 μl. Then, to stop the reaction, the samples were placed on ice, and 0.8 ml of 1.25 N NaOH was added. The formation of phenylpyruvate was analyzed at 320 nm against a control to which NaOH had been added at 0 min. The OD was read immediately after the addition of NaOH. A molar extinction coefficient of 17,500 M^-1^ cm^-1^ was used (Collier and Kohlhaw, 1972). Specific activity is defined as nanomoles of phenylpyruvate formed per minute per milligram of protein.

### 2.9 Leucine dehydrogenase assay: oxidative deamination and reductive amination

The enzyme activity was measured by spectrophotometrically monitoring the production of NADH in oxidative deamination or the consumption of NADH in reductive amination at 340 nm (*ɛ* = 6,220 M^−1^ cm^−1^). The activity was defined as the number of nanomoles of NADH produced (or consumed) in 1 min by 1 mg of enzyme (nmol min^−1^ mg^−1^).

The assay mixture for the deamination reaction contained 100 mM Tris buffer pH 9.0, 3.5 mM NAD^+^, 10 mM l-Leu (or l-Ile, l-Val) and enzyme solution in a final volume of 1 ml. The assay mixture for the reductive amination reaction contained 100 mM Tris buffer pH 7.5, 600 mM NH_4_Cl, 1 mM DTT, 0.125 mM NADH, 5 mM KIV (KMV, KIC) and enzyme solution in a final volume of 1 ml (Livesey and Lund, 1988).

### 2.10 Determination of volumetric mass transfer coefficient of oxygen (k_L_a) in test tubes cultivation conditions

The sulfite method (Cooper et al., 1944) was used to determine the volumetric mass transfer coefficient of oxygen (*k*_L_*a*) for test tubes cultivation according to Garcia-Ochoa and Gomez (2009); Cu^2+^ ions (1 mM CuSO_4_) were applied as catalyst; 1 ml samples were taken, mixed with an excess of standard iodine reagent (5 ml of 0.2N I_2_ solution) and finally titrated with tiosulfate solution (0.1N Na_2_S_2_O_3_). The measurements were performed at the range 60–120 min of incubation. The experiments were performed in triplicate.

## 3 Results and discussion

### 3.1 Construction of aminotransferase-deficient *E. coli* strain harboring feedback-resistant AHAS I

**Fig. 3:**
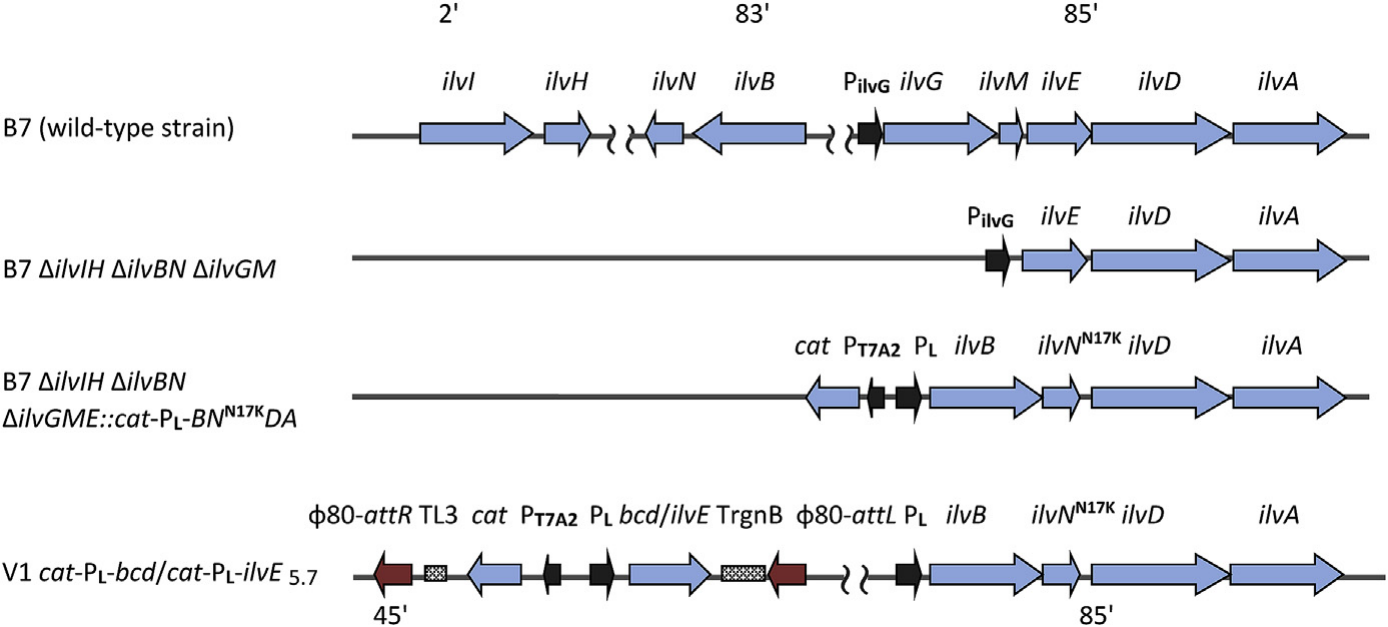
Modifications in *E. coli* chromosome. The structural parts of the genes are represented by blue arrows, the promoters are designated by black arrows, the φ80-*att* sites are represented by red arrows, the terminators are marked by shaded rectangles.

At the first step, the aminotransferase-deficient *E. coli* strain harboring feedback-resistant AHAS I was designed as a platform for the further construction of valine-producing strains overexpressing aminotransferase or, alternatively, leucine dehydrogenase genes. The AHAS-deficient *E. coli* strain K-12 B-7Δ*ilvBN* Δ*ilvGM* Δ*ilvIH* with deletions of the genes encoding acetolactate synthase types I, II and III was used as a starting point (Sycheva et al., 2009) (Fig. 3). In the native locus of the chromosome, harboring the isoleucine-valine operon (*ilv* operon, P_ilvG_-*ilvGMEDA*), this strain contains a deletion of AHAS II-encoding genes (P_ilvG_-*EDA*). To provide valine oversynthesis and, simultaneously, to inactivate the aminotransferase-encoding gene in the native locus, the operon P_ilvG_-*EDA* of this strain was replaced with the artificial construct *cat*-P_L_*-ilvBN*^N17K^*DA* harboring the feedback-resistant AHAS I-encoding genes *ilvBN*^N17K^ under control of the “strong” λ phage P_L_ promoter (Sycheva et al., 2009); the *ilvE* gene encoding aminotransferase was simultaneously deleted (for the construct design, see Materials and Methods) (Fig. 3). After elimination of the Cm^R^ marker by λ-Int/Xis site-specific recombination, the aminotransferase IlvE-deficient strain V1 (B-7Δ*ilvBN*Δ*ilvIH*Δ*ilvGME::*P_L_*-ilvBN*^N17K^*DA*) was obtained. The strain V1 is a BCAA auxotroph due to aminotransferase B (IlvE) deficiency. This strain was then used to construct the valine-producing strains carrying AT or, alternatively, LeuDH.

### 3.2 Construction of valine-producing *E. coli* strains harboring overexpressed aminotransferase or heterologous leucine dehydrogenase genes

For valine synthesis in *E. coli* under oxygen deprivation, the redox cofactor imbalance (two moles of NADH is synthesized via the EMP pathway/two moles of NADPH is consumed in valine synthesis) and the l-glutamate supply for the AT reaction are the main problems (Fig. 2). To avoid these difficulties, a replacement of the Glu-dependent aminotransferase reaction at the final step of valine biosynthesis with the NADH-dependent NH_4_^+^ assimilating dehydrogenase reaction was proposed. A range of microorganisms possess NADH-specific dehydrogenases (leucine dehydrogenase; LeuDH; EC 1.4.1.9) that carry out the reversible reductive amination of BCAA keto precursors by directly utilizing ammonia as a substrate (Livesey and Lund, 1988; Nagata et al., 1988; Ohshima et al., 1994).

Evidently, a major physiological function of these enzymes during aerobic growth on glucose is BCAA degradation (Nagata et al., 1988); however, LeuDH could be supposed to enable the amination of BCAA keto precursors and the formation of the corresponding amino acids, particularly valine, under microaerobic conditions. Considering that, in contrast to NADPH-dependent GDH, LeuDH is an NADH-dependent enzyme, the application of LeuDH seems to be preferable to the AT-exploiting metabolic pathway for the microaerobic production of valine from the viewpoint of redox cofactor balance.

#### 3.2.1 Expression of LeuDH from *B. subtilis* in *E. coli* cells

For the above purpose, in this work, LeuDH encoded by the *bcd* gene from *B. subtilis* was applied. The native nucleotide sequence of *bcd* contains a number of codons that are rare in *E. coli*; therefore, to express this LeuDH in *E. coli,* the codon-optimized variant of the *bcd* gene was chemically synthesized (see Materials and Methods) and subcloned into the integrative vector pAH162-λ*attL*-Tet^R^-λ*att*R-2Ter (Minaeva et al., 2008) for the φ80-Int-mediated insertion of the *bcd* gene into the chromosome of the wild-type strain MG1655 (see Materials and Methods). As a result, the MG1655-derived strain containing the *bcd* gene integrated into the artificial φ80-*attB* site inside the trs5-7 locus was designed by means of the φ80-Int system. After the elimination of the Tc^R^-marker-harboring part of the integrative vector by means of λ-Int/Xis site-specific recombination, the *bcd* gene was placed under control of the λ phage P_L_ promoter marked with CmR^ex^ by λRed*-*mediated recombination. As a result, the strain MG1655 *cat*-P_L_-*bcd*_5.7_ harboring the chromosomal copy of the overexpressed *bcd* gene from *B. subtilis* encoding NADH-dependent leucine dehydrogenase was obtained.

Analysis of the Bcd activity for the strain MG1655 *cat*-P_L_-*bcd*_5.7_ in the reaction of oxidative deamination revealed practically the same high level of enzymatic activity towards both substrates Val and Leu; this activity was approximately 1.8-fold higher than that using Ile as a substrate. Measurement of Bcd activity in the direction of reductive amination showed that the level of enzymatic activity was practically the same towards all the tested substrates, KIC, KIV and KMV (Tables 3 and 4).

**Table 3:** Specific leucine dehydrogenase activity in the direction of oxidative deamination in strain MG1655 *cat*-P_L_-*bcd*_5.7_.^a^

Strain	leucine dehydrogenase activity (nmol min^−1^ mg^−1^)		
	Substrate		
	l-Val	l-Leu	l-Ile
MG1655 *cat*-P_L_-*bcd*_5.7_	8270 ± 410	7900 ± 350	4410 ± 230
MG1655	11 ± 2	8 ± 2	6 ± 1

a Data represent the means of three separate experiments.

**Table 4:** Specific leucine dehydrogenase activity in the direction of reductive amination in strain MG1655 *cat*-P_L_-*bcd*_5.7_.^a^

Strain	leucine dehydrogenase activity (nmol min^−1^ mg^−1^)		
	Substrate^b^		
	KIV	KIC	KMV
MG1655 *cat*-P_L_-*bcd*_5.7_	650 ± 35	620 ± 25	560 ± 30
MG1655	6 ± 2	5 ± 1	4 ± 1

a Data represent the means of three separate experiments.

b KIV, 2-ketoisovalerate; KIC, 2-ketoisocaproate; KMV, 2-keto-3-methylvalerate.

To fulfill a similar task, valine production under oxygen deprivation in *C. glutamicum* cells, LeuDH from *Bacillus sphaericus* was previously applied (Hasegawa et al., 2012). This enzyme shares 79% amino acid sequence identity and 90% amino acid sequence positives with the LeuDH from *B. subtilis* studied in the present work. The majority of differences (23 residues) are located in domain II (residues 141–348), which resembles the classical nucleotide-binding domain of lactate dehydrogenase (Baker et al., 1995). The substrate specificity of LeuDH from *B. sphaericus* for both reductive amination and oxidative deamination was studied previously (Li et al., 2009). The substrate affinity profile for LeuDH from *B. subtilis* used in this work is similar to that reported for LeuDH from *B. sphaericus* (Li et al., 2009).

#### 3.2.2 Overexpression of ilvE gene encoding BCAA aminotransferase in *E. coli* chromosome

To provide a level of *ilvE* gene expression similar to that of the aforementioned *bcd* gene from *B. subtilis* in *E. coli* chromosome, the *ilvE* gene was inserted into the same trs5-7 locus and placed under control of the same “strong” promoter. To this end, the *ilvE* gene deletion (Δ*ilvE*::KmR^ex^) was first introduced into the chromosome of the strain MG1655 trs5-7::*bcd* by P1 transduction, yielding the BCAA auxotrophic ilvE-deficient strain MG1655 Δ*ilvE*::KmR^ex^ trs5-7::*bcd*. Notably, in this case, the promoter-less *bcd* gene did not provide growth on minimal medium in the absence of BCAAs. Then, the PCR fragment λ*attL*-Cm^R^-λ*attR*-P_L_-*ilvE,* containing the *ilvE* gene under the control of the λ phage P_L_ promoter, was directly inserted into the chromosome of this strain in place of the *bcd* gene by λRed*-*mediated recombination. Integrants were selected on M9 minimal medium with 0.4% glucose to obtain the strain MG1655 *cat*-P_L_-*ilvE*_5.7_ Δ*ilvE*::Km^R^ harboring the aminotransferase gene under the control of the “strong” λP_L_ promoter.

The obtained chromosomal construct *cat*-P_L_-*ilvE*_5.7_ was shown to provide essentially higher (more than 100-fold) BCAA TA activity than the native copy of the same gene (Table 5). KIV, an immediate precursor of valine, was used as a substrate in these experiments.

**Table 5:** Specific BCAA aminotransferase activity in strain MG1655 *cat-*P_L_*-ilvE*_5.7_ Δ*ilvE*::Km^R^.

Strain	BCAA aminotransferase activity (nmol min^−1^ mg^−1^)
	KIV^a^
MG1655 *cat-*P_L_*-ilvE*_5.7_	1060 ± 45
MG1655	4 ± 0.5

a KIV- 2-ketoisovalerate as a substrate; data represent the means of three separate experiments.

To measure the efficiency in *E. coli* of the native valine biosynthetic pathway, including NADPH-dependent aminotransferase-mediated valine formation and the artificial one, including NADH-dependent leucine dehydrogenase-mediated NH_4_^+^ assimilation at the final step, we designed valine-producing strains containing one chromosomal copy of the *bcd* gene or the *ilvE* gene. The aminotransferase IlvE-deficient strain V1 (B-7 Δ*ilvBN* Δ*ilvIH* Δ*ilvGME*::P_L_*-ilvBN*^*N17K*^*DA)* was used as the recipient for construction, as it can produce Val upon restoration its ability to perform the last step of Val synthesis, 2-ketoisovalerate (KIV) amination. To this end, the expression units *cat-*P_L_*-bcd*_5.7_ and *cat-*P_L_*-ilvE*_5.7_ were separately introduced into the chromosome of V1 by P1 transduction, resulting in the strains V1 *cat-*P_L_*-bcd*_5.7_ and V1 *cat-*P_L_*-ilvE*_5.7_ (Fig. 3). Both of these strains demonstrated similar growth on M9 plates with 0.4% glucose in the absence of Val, Ile and Leu addition, which indicated the ability of the heterologous leucine dehydrogenase Bcd from *B. subtilis* to enable the *in vivo* formation of valine by *E. coli* cells.

### 3.3 Valine accumulation by valine-producing *E. coli* strains under different cultivation conditions

LeuDH effectively catalyzes the reversible reductive amination of BCAA keto precursors using NADH as a cofactor and ammonia as a substrate (Fig. 1). Therefore, the introduction of LeuDH in place of native AT should improve the intracellular redox balance by reoxidizing NADH and, in the case of a sufficient NH_4_^+^ supply, increase the valine production under deprivation conditions. We evaluated the obtained strains V1 *cat-*P_L_*-bcd*_5.7_ and V1 *cat-*P_L_*-ilvE*_5.7_ under different cultivation conditions, aerobic and microaerobic, for 68 h at 32 °C (Table 6). As shown in Table 6, the expression of leucine dehydrogenase Bcd or aminotransferase IlvE in strain V-1 (B-7 Δ*ilvBN* Δ*ilvIH* Δ*ilvGME*::P_L_*-ilvBN*^*N17K*^*DA*) in both cases resulted in the production of valine.

**Table 6:** Valine accumulation by Bcd- or IlvE- harboring *E. coli* strains under different aeration conditions.^a^

Strain	O_2_ conditions^b^	Val, g/L	OD_540_	Glc residual, g/L	Y m/m, %
V1 *cat*-P_L_-*bcd*_5.7_	microaerobic	9.1 ± 0.3	19.5 ± 0.3	20.3 ± 0.4	35.3 ± 0.8
	aerobic	6.9 ± 0.1	34.5 ± 0.8	0	17.7 ± 0.3
V1 *cat*-P_L_-*ilvE*_5.7_	microaerobic	4.1 ± 0.1	14.5 ± 0.3	24.6 ± 0.3	17.8 ± 0.3
	aerobic	9.8 ± 0.4	31.3 ± 1.8	0	25.1 ± 1.1

a Cultivation time was 48 h; data represent the means of three separate experiments; *k*_L_a was determined by sulfite method as described in p2.10 “Materials and methods”.

b Microaerobic conditions (*k*_L_a = 5.4 ± 0.2 × 10^−4^, mmole _O2_ ml^−1^ min^−1^); aerobic conditions (*k*_L_a = 14.2 ± 0.4 × 10^−4^, mmole _O2_ ml^−1^ min^−1^).

**Fig. 4:**
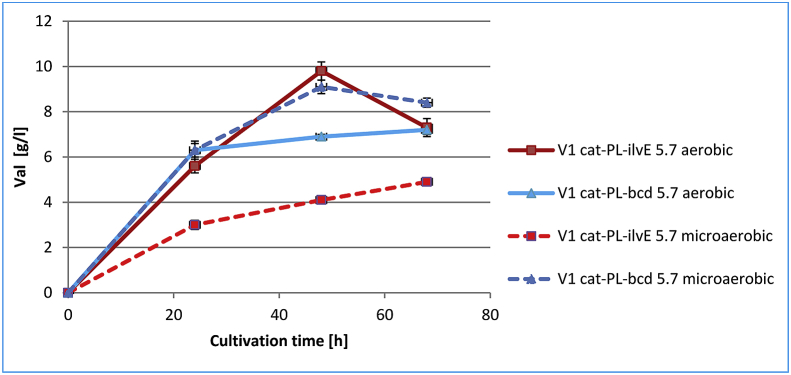
Time-course profiles of valine accumulation by Bcd- or IlvE- harboring *E. coli* strains under different aeration conditions. Three independent cultivations were performed.

**Fig. 5:**
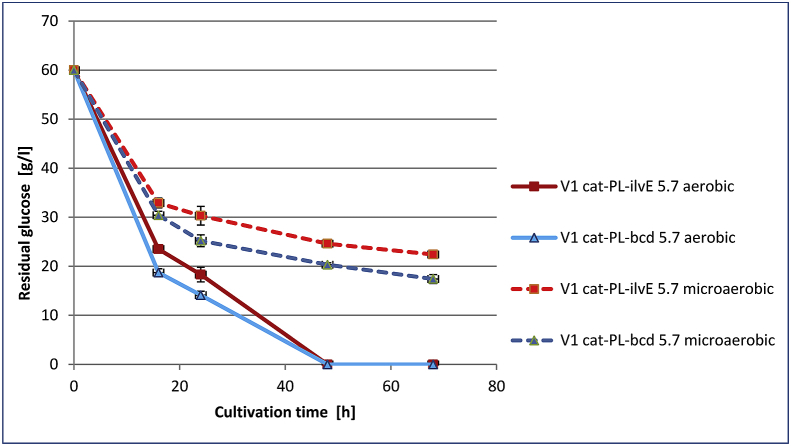
Time-course profiles of glucose consumption by Bcd- or IlvE- harboring *E. coli* strains under different aeration conditions. Three independent cultivations were performed.

**Fig. 6:**
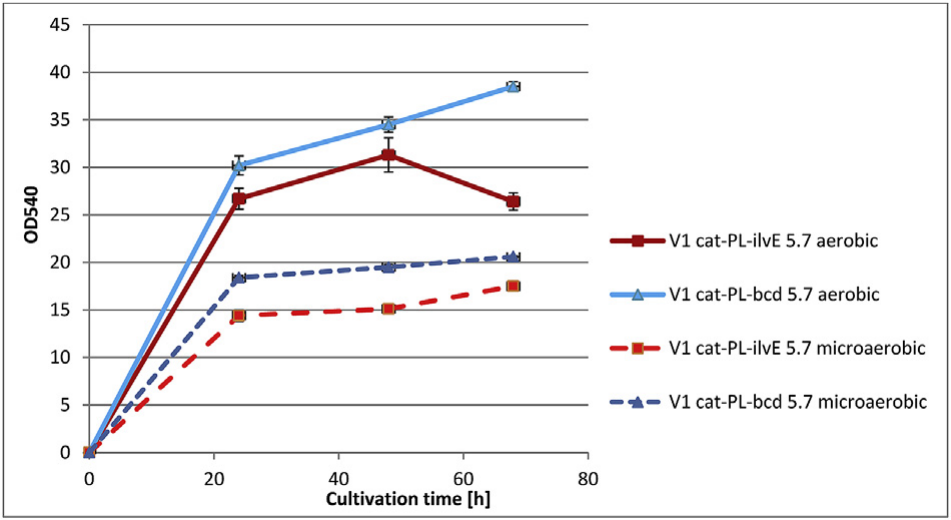
Time-course profiles of cell growth for Bcd- or IlvE- harboring *E. coli* strains under different aeration conditions. Three independent cultivations were performed.

Time-course profiles of Val accumulation, glucose consumption and cell growth were analyzed for the strains V1 *cat-*P_L_*-bcd*_5.7_ and V1 *cat-*P_L_*-ilvE*_5.7_ under microaerobic and aerobic conditions (Figs. 4, 5, and 6). For both the tested strains glucose was completely consumed at 48h under aerobic conditions in contrast to that under microaerobic conditions. The maximum level of Val accumulation was observed for tested strains V1 *cat-*P_L_*-bcd*_5.7_ and V1 *cat-*P_L_*-ilvE*_5.7_ at 48h under microaerobic and aerobic conditions, respectively (Fig. 4).

Under microaerobic cultivation conditions, the Bcd-possessing strain V1 *cat-*P_L_-*bcd*_5.7_ accumulated 2.2-fold more valine than the IlvE-possessing strain V1 *cat*-P_L_-*ilvE*_5.7_. Additionally, decreasing the oxygen supply resulted in a 32% increase in Val accumulation by the Bcd-possessing strain V1 *cat-*P_L_-*bcd*_5.7_ compared with that in aerobic conditions. Thus, the Bcd-possessing strain was capable of the efficient synthesis of valine under both aeration conditions. In contrast, Val production by the IlvE-possessing strain V1 *cat*-P_L_-*ilvE*_5.7_ was decreased by 2.4 times under microaerobic cultivation conditions, which clearly indicated a drawback of the AT-mediated step limited by an insufficient supply of l-glutamate. A decrease in optical density (∼2-fold) and a significant amount of residual glucose were observed under microaerobic cultivation conditions for both the tested strains. Under these conditions, the Bcd-containing strain accumulated Val with a 2.0-fold increased yield (Y, m/m) over that of V1 *cat*-P_L_-*ilvE*_5.7_. Additionally, the Bcd-possessing strain accumulated Val with a 1.4-fold increased yield under microaerobic conditions compared to that of V1 *сat*-P_L_-*ilvE*_5.7_ under aerobic conditions, but Val accumulation was decreased by 7.1%. LeuDH catalyzed the amination of KIV much more efficiently than AT under microaerobic conditions. The IlvE-possessing strain showed higher Val accumulation (9.8 g/L) under aerobic conditions, which can be explained by the level of NADPH in the cells.

The application of LeuDH instead of AT at the final step of valine biosynthesis altered the amino group donor and cofactor requirement for valine synthesis from l-glutamate and NADPH to NH_4_^+^ and NADH, respectively (Fig. 2), which increased both valine accumulation and yield under microaerobic conditions. In this case, a sufficient NH_4_^+^ supply and sufficient intracellularly accumulated NADH are the main requirements for efficient valine synthesis by LeuDH.

The obtained results are in agreement with the data obtained by the application of a similar approach in *C. glutamicum* (Hasegawa et al., 2012, 2013), where the improvement of the redox cofactor balance in valine synthesis by the replacement of NADPH-dependent reactions (AT and KARI) with NADH-dependent reactions resulted in increased Val accumulation, yield and glucose consumption rate.

The Bcd-containing *E. coli* strain used in our experiments possessed unmodified pathways for mixed fermentation (lactate, ethanol, etc.); therefore, further steps in the improvement of valine production under microaerobic conditions should include the restriction of these pathways’ functioning to make “valine fermentation” the main route for the required NADH oxidation. Additionally, changes in the KARI reaction cofactor specificity from NADPH to NADH and/or enhancement of PntAB transhydrogenase functioning seem promising for further improvement of the redox cofactor balance in the process of valine production under microaerobic conditions.

## 4 Conclusions

LeuDH from different species is widely used for the synthesis of a range of compounds by biotransformation, such as l-ABA from threonine (Тао et al., 2014) and l-*tert*-leucine from trimethylpyruvate (TMP) (Zhu et al., 2016). Additionally, NADH-dependent LeuDH from *B. sphaericus* was used instead of endogenous NADPH-dependent AT for successful valine synthesis in *C. glutamicum* under oxygen deprivation (Hasegawa et al., 2012, 2013)*.* l-Alanine synthesis was shown under oxygen deprivation conditions in *C. glutamicum* by using the alanine dehydrogenase AlaD from *B. sphaericus* (Jojima et al., 2010). Changing the cofactor requirements from NADPH to NADH in AA biosynthesis resulted in increased accumulation and glucose consumption rates under oxygen deprivation in *C. glutamicum* (Hasegawa et al., 2012, 2013; Jojima et al., 2010). The efficient isobutanol synthesis in *E. coli* under anaerobic conditions at theoretical yield by using the NADH-dependent pathway was shown (Bastian et al., 2011).

Microaerobic conditions may be preferable for the production of valine considering that pyruvate, generated by the EMP pathway, is simultaneously a starting compound for the synthesis of this amino acid and a substrate for pyruvate dehydrogenase (PDH), which involves pyruvate in the respiratory process. At the same time, AHASes, which are responsible for the first step of valine synthesis from pyruvate, cannot compete with PDH for this substrate due to their approximately two orders of magnitude lower affinity (Bisswanger, 1981). Thus, limiting PDH activity by cultivation under oxygen deprivation conditions seems promising for valine production.

In this study, we have shown the application of leucine dehydrogenase Bcd from *B. subtilis* for valine synthesis in *E. coli* under microaerobic conditions. We have demonstrated the effective synthesis of valine by means of so-called “valine fermentation” (Fig. 1). The Bcd-possessing valine-producing strain containing one chromosomal copy of the artificial operon P_L_-*ilvBN*^*N17K*^*DA* was able to accumulate a 2.2-fold higher amount of valine with a 2.0-fold increased yield (m/m) compared with the IlvE-possessing strain under microaerobic cultivation conditions*.* Additionally, the Bcd-possessing strain accumulated Val with 1.4-fold increased yield (m/m) under microaerobic conditions compared to that of the IlvE*-*possessing strain under aerobic conditions, although Val accumulation was decreased by 7.1%. Thus, microaerobic fermentation can be favorable as an economical, environmentally friendly process for production at scale with high yield.

## Declarations

### Author contribution statement

Ekaterina A. Savrasova: Conceived and designed the experiments; Performed the experiments; Analyzed and interpreted the data; Wrote the paper.

Nataliya V. Stoynova: Conceived and designed the experiments; Analyzed and interpreted the data; Contributed reagents, materials, analysis tools or data; Wrote the paper.

### Funding statement

This research did not receive any specific grant from funding agencies in the public, commercial, or not-for-profit sectors.

### Competing interest statement

The authors declare no conflict of interest.

### Additional information

No additional information is available for this paper.
